# Aspartate aminotransferase-lymphocyte ratio index and systemic immune-inflammation index predict overall survival in HBV-related hepatocellular carcinoma patients after transcatheter arterial chemoembolization

**DOI:** 10.18632/oncotarget.5719

**Published:** 2015-10-16

**Authors:** Zongguo Yang, Jianliang Zhang, Yunfei Lu, Qingnian Xu, Bozong Tang, Qiang Wang, Wensi Zhang, Shishi Chen, Lingqing Lu, Xiaorong Chen

**Affiliations:** ^1^ Shanghai Public Health Clinical Center, Fudan University, Shanghai 201508, China

**Keywords:** lymphocyte cell, aspartate aminotransferase-lymphocyte ratio index, systemic immune-inflammation index, survival, hepatocellular carcinoma

## Abstract

It has been suggested that lymphocytes play central roles in host antitumor immune responses and control cancer outcome. We reviewed the clinical parameters of 189 hepatocellular carcinoma (HCC) patients and investigated the prognostic significance of lymphocyte-related scores in HCC patients following transcatheter arterial chemoembolization (TACE). Survival analysis revealed that an elevated aspartate aminotransferase-lymphocyte ratio index (ALRI) > 57 and a systemic immune-inflammation index (SII) > 300 were negatively associated with overall survival in HBV-related HCC (HR = 2.181, *P* = 0.003 and HR = 2.453, *P* = 0.003; respectively). Spearman chi-square analysis showed that ALRI had a specificity of 82.4% and that SII index had a sensitivity of 71.9% for HCC overall survival. ALRI and SII had negative predictive values of 74.6% and 80%, respectively for HCC overall survival. Additionally, Barcelona Clinic Liver Cancer (BCLC) stage C patients had significantly higher ALRI and SII scores (both *P* < 0.0001) and poorer overall survival (HR = 3.618, *P* < 0.001). Additionally, HCC patients with portal vein tumor thrombosis (PVTT) had higher ALRI and SII scores (*P* < 0.0001 and *P* = 0.0059, respectively). In conclusion, as noninvasive, low cost, easily assessable and reproducible parameters, elevated ALRI and SII should be used as negative predictive factors for overall survival in HBV-related HCC in clinical practice.

## INTRODUCTION

Recent epidemiologic data have revealed that liver cancer may account for more cancer-related deaths globally than previously reported [1–3]. Currently, there are an estimated 300 million carriers of HBV worldwide, and as many as 25% of those carriers may develop HCC [4, 5]. The widespread search for effective biomarkers of HCC is aimed at achieving earlier diagnosis and improving prognosis by allowing for earlier intervention [6].

Systemic inflammatory responses have been shown to reflect the promotion of angiogenesis, DNA damage and tumor invasion through upregulation of cytokines [7–9]. Previous research revealed that lymphocytes play a crucial role in tumor defense by inducing cytotoxic cell death and inhibiting tumor cell proliferation and migration [10]. Research has also demonstrated that patrolling and infiltrating lymphocytes reflect the host's inflammatory status as well as the ability of the host's body to exert decisive antitumor immune responses [11, 12]. In consideration of these factors, several inflammation and immune-based prognostic scores, such as lymphocyte count, neutrophil-lymphocyte ratio (NLR), and systemic immune-inflammation index (SII), have been developed to predict survival and recurrence in cancers, including HCC [13–15]. Recently, a novel index that combines aspartate aminotransferase (AST) and lymphocyte count was used to predict the prognosis of HCC [16]. Nevertheless, conflicting data have emerged regarding the ability of some prognostic scores, such as NLR, to predict disease progression and overall survival in HCC [14].

This study evaluated the efficacy of lymphocyte-related inflammation and immune-based prognostic scores, including ALRI, monocyte-lymphocyte ratio (MLR), NLR, platelet-lymphocyte ratio (PLR) and SII, as predictors of overall survival in HCC patients following transcatheter arterial chemoembolization (TACE). These data may provide new insights into the use of these biomarker candidates for the determination of the pathogenesis, progression and prognosis of HCC.

## RESULTS

### Patient characteristics

As shown in Table 1, a total of 189 HCC patients were included in this study: 64 nonsurvivors and 125 survivors. Among the liver function values tested, the levels of AST, total bilirubin (TBiL), gamma-glutamyl transferase (GGT) and lactate dehydrogenase (LDH) were statistically higher in the nonsurvivor group than in the survivor group (all *P* < 0.05). Furthermore, HCC nonsurvivors had a lower albumin level and longer prothrombin time (both *P* < 0.05). More of the HCC nonsurvivors suffered from ascites than the HCC survivors (*P* = 0.005). The Child-Pugh stage and BCLC stage distributions were different between these two groups (both *P* < 0.05). In addition, more patients had evidence of portal vein tumor thrombosis (PVTT), inferior vena cava invasion and metastasis in the HCC nonsurvivor group (all *P* < 0.05). The median alpha-fetoprotein (AFP) level was significantly higher in the nonsurvivor group than in the survivor group (*P* < 0.001), and the median follow-up period was longer in the HCC survivor group than in the nonsurvivor group (*P* < 0.001). The distribution of the other baseline characteristics of the HCC patients was not different between these two groups.

**Table 1:** Baseline characteristics of HCC patients

Characteristics	Survival, *n* = 125	Death, *n* = 64	*P* value
Age, years	53.8 ± 10.6	52.6 ± 10.2	0.693
Male, *n*	103	58	0.132
ALT, U/L	36 (5–456)	34 (11–335)	0.917
AST, U/L	39 (15–603)	55 (16–430)	0.001
TBiL, μmol/L	14.4 (5.6–110.8)	17.1 (8.3–90.1)	0.019
GGT, U/L	90 (15–1196)	149 (19–838)	0.004
LDH, U/L	188 (107–733)	225 (136–1037)	0.001
Albumin, g/L	39.7 ± 4.9	37.3 ± 4.9	0.002
Prothrombin time, seconds	12.5 (11.3–17.0)	12.8 (10.9–15.3)	0.041
Ascites, *n*	5	10	0.005
Child-pugh stage, *n*			0.002
A	116	49	
B and C	9	15	
Cirrhosis, *n*	107	58	0.326
Tumor size >5 cm, *n*	31	22	0.166
Multicentric tumor, *n*	47	31	0.152
Portal vein tumor thrombosis, *n*	30	29	0.003
Metastasis, *n*	22	29	<0.001
BCLC stage			<0.001
A	9	0	
B	74	15	
C	42	49	
Tumor location, *n*			0.285
Left lobe	22	14	
Right lobe	66	26	
Left and right lobes	37	24	
Inferior vena cava invasion, *n*	8	10	<0.001
Diabetes mellitus, *n*	15	6	0.587
Hypertension, *n*	21	7	0.283
Alpha fetoprotein, ng/ml	174 (1–3630)	3322 (2–3630)	<0.001
History of hepatectomy, *n*	29	14	0.837
Tumor differentiation, *n*			1.0
I-II	12	4	
III-IV	9	3	
Follow-up period, months	19.8 (0.2–98.3)	7.6 (0.8–71.3)	<0.001

### Association of ALRI and SII with HCC overall survival

Table 2 summarizes the results of the univariate Cox regression analyses of factors associated with HCC overall survival. *R* software was used to randomly select 94 HCC patients as a training set and 95 HCC patients as a validation set. All liver function parameters, clinico-pathological features, blood routine parameters, and lymphocyte-related indexes, including ALRI, MLR, NLR, PLR and SII, were included in the Cox univariate analyses. In both sets, the following factors were significantly associated with HCC overall survival: lymphocyte count, ALRI, MLR, NLR, PLR, SII, AST, GGT, LDH, PVTT, BCLC stage, inferior vena cava invasion and metastasis (all *P* < 0.10, Table 2).

**Table 2:** Univariate Cox regression analyses of the parameters of blood routine tests and overall survival from HCC

Variables	Training set, *n* = 94		Validation set, *n* = 95	
	HR (95% CI)	*P* value	HR (95% CI)	*P* value
Lymphocyte cells counts, per increase of 1000 cells/mm^3^	0.477 (0.227–1.002)	0.051	0.546 (0.281–1.062)	0.075
ALRI, per increase of 1 unit	1.005 (1.001–1.008)	0.015	1.004 (1.001–1.006)	0.001
MLR, per increase of 1 unit	12.932 (1.522–109.883)	0.019	3.821 (1.434–10.178)	0.007
NLR, per increase of 1 unit	1.521 (1.185–1.953)	0.001	1.208 (1.102–1.324)	<0.001
PLR, per increase of 1 unit	1.008 (1.003–1.013)	0.002	1.006 (1.003–1.009)	<0.001
SII, per increase of 1 unit	1.001 (1.0–1.001)	0.025	1.001 (1.0–1.001)	0.002
AST, per increase of 1 unit	1.005 (1.001–1.01)	0.008	1.003 (1.001–1.006)	0.016
GGT, per increase of 1 unit	1.001 (1.0–1.003)	0.073	1.001 (1.0–1.003)	0.025
LDH, per increase of 1 unit	1.004 (1.002–1.005)	<0.001	1.003 (1.001–1.004)	<0.001
PVTT, yes vs. no	2.985 (1.382–6.448)	0.005	2.76 (1.413–5.392)	0.003
BCLC stage, BCLC stage C vs. stage A-B	4.497 (1.896–10.666)	0.001	6.371 (2.832–14.332)	<0.001
Inferior vena cava invasion, yes vs. no	4.042 (1.455–11.228)	0.007	2.635 (1.073–6.472)	0.035
Metastasis, yes vs. no	2.742 (1.284–5.855)	0.009	2.716 (1.388–5.314)	0.004

Furthermore, we performed *R* software analysis to determine the cut-off values of lymphocyte count, ALRI, MLR, NLR, PLR and SII for the prediction of overall survival based on the data in the training set. Then, we transformed the continuous data above into dichotomous variables according to the determined cut-off values. The factors significantly associated with HCC overall survival in both the training and validation sets as determined by the univariate Cox regression analyses were included in the multivariate Cox regression analyses. As shown in Table 3, when these factors were evaluated in a multivariate model using forward selection, ALRI (HR = 2.181, 95% CI = 1.304–3.648, *P* = 0.003) and SII (HR = 2.453, 95% CI = 1.353–4.446, *P* = 0.003) were significantly associated with HCC overall survival. Consistent with previous reports, higher BCLC stage was also associated with poorer overall survival in HCC (HR = 3.618, 95% CI = 1.973–6.638, *P* < 0.001). No significant association between MLR, NLR, PLR or lymphocyte count and overall survival was found in the HCC patients.

**Table 3:** Multivariate Cox regression analyses in the training and validation sets (*n* = 189)

Variables	Category	HR (95% CI)	*P* value
ALRI	>57 vs. <57	2.181 (1.304–3.648)	**0.003**
SII	>300 vs. <300	2.453 (1.353–4.446)	**0.003**
BCLC stage	stage C vs. stage A-B	3.618 (1.973–6.638)	**<0.001**

NS, no significance.

**Figure 1:**
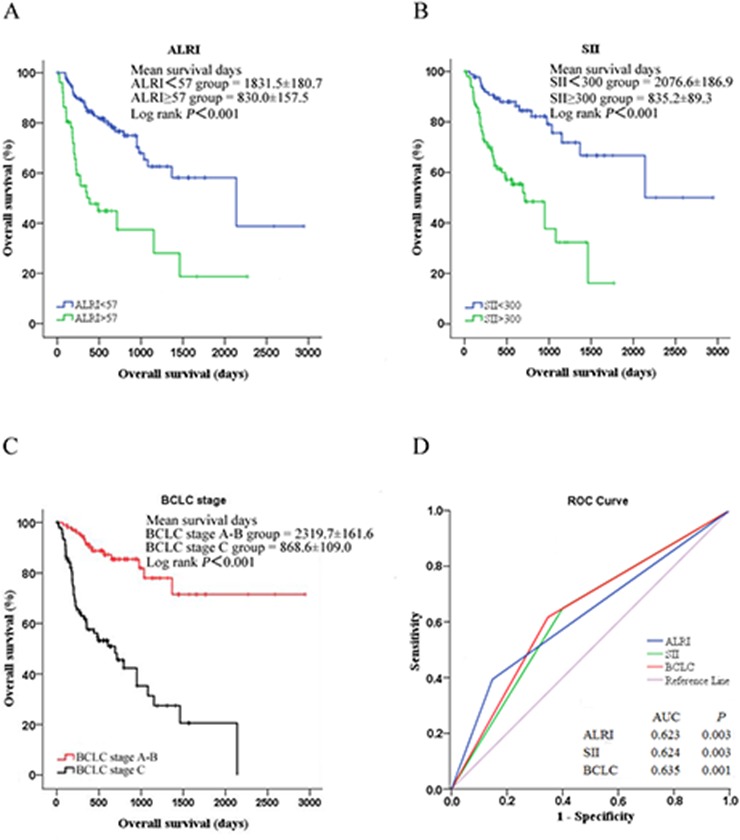
Analysis of HCC overall survival by **A.** ALRI, **B.** SII and **C.** BCLC stage using the Kaplan-Meier survival method; **D.** ROC curves of ALRI, PLR and SII for HCC overall survival, with a median survival time of 524 days.

Additionally, we performed a Kaplan-Meier event analysis of the factors identified to be significantly associated with survival above. We grouped the ALRI and SII indexes using cut-off values of 57 and 300, respectively, into normal groups and elevated groups. This revealed that elevated ALRI and SII indexes significantly negatively impacted HCC overall survival (both log-rank *P* < 0.001, Figure 1A and 1B). Additionally, BCLC stage C patients had poorer overall survival than BCLC stage A and B patients (log-rank *P* < 0.001, Figure 1C).

### Prognostic values of ALRI and SII for HCC overall survival

To evaluate the predictive accuracy of serum ALRI and SII indexes for HCC overall survival, we analyzed ROCs and found that elevated ALRI, SII and BCLC stage significantly and accurately predicted HCC overall survival (AUC = 0.623, 0.624 and 0.635, respectively, all *P* < 0.01, Figure 1D). Moreover, we conducted Spearman chi-square analysis to evaluate the prognostic values of ALRI and SII for HCC overall survival. As shown in Table 4, ALRI had a specificity of 82.4%, and the SII index had a sensitivity of 71.9% for the prediction of overall survival. ALRI and SII had 74.6% and 80% negative predictive values for HCC overall survival. Unfortunately, ALRI and SII had accuracies below 70%. Well-designed studies with large sample sizes are needed to reevaluate the prognostic roles of these factors in HCC patients.

**Table 4:** Predictive value of ALRI and SII for HCC overall survival

Indexes	Cut-off	AUC (95% CI)	Sensitivity	Specificity	PV_+_	PV_−_	LR_+_	LR_−_	Accuracy	*P* value
ALRI	57	0.639 (0.552–0.725)	45.3	82.4	56.9	74.6	2.6	0.7	69.8	0.002
SII	300	0.647 (0.565–0.729)	71.9	57.6	46.5	80.0	1.7	0.5	62.4	0.001

AUC, area under curve; PV_+_, positive predictive value; PV_−_, negative predictive value; LR_+_, positive likelihood ratio; LR_−_, negative likelihood ratio.

### Comparison of ALRI and SII indexes in different HCC subgroups

**Figure 2:**
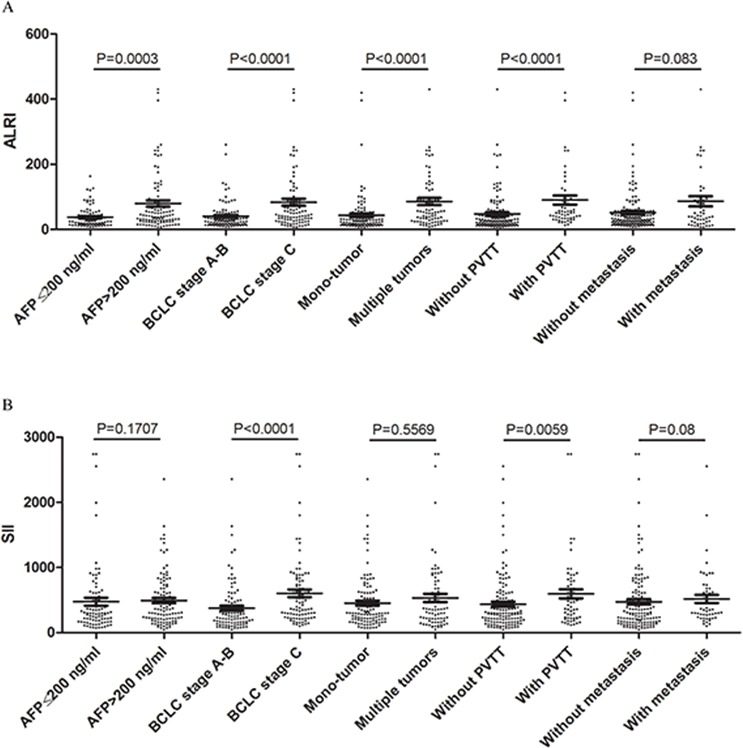
Comparisons of **A.** ALRI and **B.** SII in different HCC subgroups, including AFP level, BCLC stage, tumor number, PVTT and metastasis.

It is well known that clinico-pathological features, including AFP level, BCLC stage, tumor number, PVTT and metastasis, are risk factors associated with HCC survival. Thus, we compared ALRI and SII indexes in subgroups with these different clinico-pathological features, and the results are summarized in Figure 2. HCC patients with an AFP level over 200 ng/ml, higher BCLC staging, multiple tumors and/or PVTT had higher ALRI scores (all *P* < 0.001, Figure 2A). HCC patients with BCLC stage C and/or PVTT had significantly higher SII scores than those with BCLC stage A to B without PVTT (*P* < 0.0001 and *P* = 0.0059, respectively, Figure 2B). ALRI and SII scores were elevated in HCC patients with BCLC stage C and/or PVTT. Therefore, we propose that ALRI and SII may be associated with HCC aggressiveness and progression.

## DISCUSSION

Hepatocellular carcinoma (HCC) is one of the most common potentially lethal human malignancies worldwide [17]. Few tumor markers have been externally validated for their value in HCC survival prediction [16, 18]. Therefore, it is of great importance to find convenient, easily-obtained, low cost, reliable and non-invasive biochemical markers for HCC prognosis prediction.

Early in the neoplastic process, inflammatory cells are powerful tumor promoters; they produce an attractive environment for tumor growth, facilitating genomic instability and promoting angiogenesis [19–21]. Tumors are often infiltrated by various numbers of lymphocytes, macrophages and mast cells. It has been suggested that lymphocytes play central roles in host antitumor immune responses. Mouse models have shown that lymphocytes may control cancer outcome [22]. In our research, Cox univariate and multivariate analyses demonstrated that ALRI and SII were significantly associated with HCC overall survival. These results were also confirmed by Kaplan-Meier analysis using log-rank methods.

As a simple non-invasive prognostic marker, ALRI was first described by Jin et al [16] in HCC patients after hepatic resection. Jin et al [16] concluded that preoperative ALRI was an independent prognostic factor for disease-free survival and overall survival in HCC patients. Additionally, preoperative ALRI also showed differing prognostic value in various subgroups of HCC. Partially consistent with previous results, HCC patients with an AFP level more than 200 ng/ml, higher BCLC staging, multiple tumors and/or PVTT had higher ALRI scores. It is well known that AST is a reliable and sensitive biochemical marker of liver injury. A higher AST level is correlated with a greater influx of HBV, which is associated with decreased overall survival in HCC patients. Previous studies have demonstrated that AFP level, BCLC stage, multicentric tumors and PVTT are all factors associated with HCC survival [4, 23–25]. Considering the results above, we propose that ALRI is a noninvasive, simple, and easily assessable independent effective predictor of prognosis for patients with HCC. However, the cut-off value of ALRI in our report was different from that previously reported [16]. Prospective studies with large samples should be conducted to justify our findings regarding the value of ALRI for HCC prognosis.

As an integrated indicator based on peripheral lymphocyte, neutrophil, and platelet counts, the predictive value of SII for cancer outcomes might be due to the function of these three types of cells [15]. Lymphocytes and platelets have been proven to promote tumor development. In addition, recent evidence indicates that neutrophils enhance cancer cell invasion, proliferation, and metastasis and assist cancer cells with evading immune surveillance [26, 27]. Hence, SII is a promising independent predictive factor for the prognosis of patients with HCC. In our analysis, we found that elevated SII with a cut-off value of 300 was negatively associated with HCC overall survival. Moreover, elevated SII was associated with BCLC staging and PVTT, which both indicate a more aggressive phenotype. Fan J et al [15] also found that patients with an elevated preoperative SII were usually diagnosed with thrombocytopenia, neutrophilia, or lymphopenia, suggesting an elevated inflammatory status and weak immune response. They believed that a better understanding of the role of neutrophils, platelets, and lymphocytes in cancer would help clarify the association between cancer, immunity, and inflammation. The potential mechanism through which elevated SII is associated with poorer outcome in HCC is an increase in the dissemination of tumor cells via the circulation, allowing tumor cells to escape immune surveillance and increasing the level of peripheral circulating tumor cells [15].

The limitations of this study include its retrospective nature and small sample size, which generate potential biases. Second, ALRI and SII did not have powerful prognostic values for HCC overall survival in our study. Therefore, we suggest that they be used in combination in clinical practice. Third, some clinico-pathological characteristics, including capsule state, portal hypertension and TNM stage, were not available in our report. Thus, further prospective, well-designed studies with larger samples focused on the relationship between inflammation cell-related indexes and HCC prognosis should be conducted.

In summary, as noninvasive, low cost, easily assessable and reproducible parameters, ALRI and SII are promising tools for the assessment of HCC prognosis in clinical practice in the future. Currently, it is clear that anti-inflammatory therapy is effective at preventing early neoplastic progression and malignant conversion [19]. A better understanding of the roles of inflammatory cells in cancer will help elucidate the association between cancer, immunity, and inflammation, leading to promising anti-cancer treatments and improved prognosis. The mechanism behind the associations of elevated ALRI and elevated SII with poorer prognosis in HCC patients should be clarified.

## MATERIALS AND METHODS

### Ethics statement

The survey protocol of this study was in accordance with the ethical guidelines of the Declaration of Helsinki. All participants provided written informed consent during their hospitalization. The study protocol and informed consent documents were reviewed and approved by the Ethics Committee of Shanghai Public Health Clinical Center, Fudan University.

### Patients

We retrospectively reviewed the medical records of all HCC patients who visited Shanghai Public Health Clinical Center from September, 2009 to May, 2015 to confirm eligibility based on the relevant clinico-pathological parameters. The diagnosis of HCC was either verified pathologically or diagnosed on the basis of radiologic criteria according to European Association for the Study of the Liver - two imaging studies, including computed tomography (CT) or magnetic resonance imaging (MRI), showing an arterial enhanced mass greater than 2 cm or one imaging study (CT or MRI) showing an arterial enhanced mass greater than 2 cm and an AFP level greater than 400 ng/ml [28]. All of the participants had no other lymphatic system disorders or malignant hematologic diseases, ensuring that the whole blood parameters were representative of normal baseline values. Patients with renal and/or hepatic failure, acute coronary syndromes, valvular heart diseases, autoimmune thyroid diseases, or systematic inflammatory diseases were excluded from our study.

### Study design

This was a retrospective, single-center study. The baseline features, including demographics, tumor markers, routine blood test results, liver function parameters and surgical resection history, were all extracted. The following variables were collected for the analysis: age and gender of the patient; date of HCC diagnosis and date of death or last follow-up; presence of cirrhosis, diabetes, hypertension, and ascites; the main serological parameters, including alanine aminotransferase (ALT), AST, TBiL, GGT, LDH, albumin and prothrombin time; tumor characteristics (number of lesions, portal vein tumor thrombosis, inferior vena cava invasion, tumor location, metastasis, node status, and tumor differentiation) and surgical resection history.

### Definition

The routine blood and liver function tests performed within one week before TACE were considered in this analysis. Five inflammatory factors, including ALRI, MLR, NLR, PLR and SII were included in this analysis. The definitions of these factors are as follows: ALRI = *AST / L*; MLR = *M / L*; NLR = *N / L*; PLR = *P / L*; and SII = *P* × *N / L*, where *M, L, N* and *P* are the peripheral monocyte, lymphocyte, neutrophil and platelet counts, respectively.

All baseline laboratory tests were performed at the Department of Laboratory Medicine, Shanghai Public Health Clinical Center. Following the Standard Operation Procedure (SOP) of our laboratory department, the elapsed time between obtaining the blood samples and performing the routine blood and liver function tests were 30 minutes and 1 hour, respectively. The Modular analytics P800 (Roche Diagnostics, Shanghai, China) and Sysmex XT-4000i (Sysmex Corporation, Kobe, Japan) were used to measure the liver function tests and whole blood parameters, respectively. The reference ranges of the liver function parameters were as follows: ALT, 10 to 40 U/L; AST, 10 to 40 U/L; TBiL, 0 to 17.1 umol/L; GGT, 10 to 60 U/L; LDH, 109 to 245 U/L; albumin, 35 to 55 g/L; prothrombin time, 11.0 to 13.7 seconds; and AFP, less than 7.0 ng/ml.

### Follow-up

We obtained the patients' follow-up data by reviewing inpatient and outpatient medical records and directly from follow-up visits. Overall survival was defined as the time from HCC diagnosis until death due to any cause. Tumor stage was established according to the Barcelona Clinic Liver Cancer (BCLC) stage system. Child-Pugh score was calculated based on the severity of hepatic encephalopathy, ascites, total bilirubin, albumin, and prothrombin time [29]. Tumor characteristics and liver histology status, including cirrhosis, a multicentric tumor, portal vein tumor thrombosis, inferior vena cava invasion, tumor location and metastasis, were diagnosed by imaging studies, such as CT/MRI.

### Statistical analysis

Student's *t*-test was used to compare mean values for normally distributed continuous data, and the Mann-Whitney *U*-test was used to compare mean values for non-normally distributed continuous data. Factors associated with HCC overall survival were assessed by both Cox univariate and multivariate analyses. Only covariates significantly associated with outcomes in the univariate analysis (two-sided *P* < 0.10) were included in the multivariate model. *R* software was used to analyze the results of the regression analyses to determine the cut-off values of the factors associated with HCC overall survival. The results are reported as hazard ratios (HR) with 95% confidence intervals (CI). The Kaplan-Meier method was used to compare overall survival between different groups, and the log-rank test was used to estimate the differences in survival between groups. ROC analysis was performed to evaluate predictive values of potential factors for HCC survival. The statistical analyses were performed using PASW Statistics software version 18.0 from SPSS Inc. (Chicago, IL, USA). All statistical tests were two-tailed, and differences with *P* < 0.05 were considered statistically significant.
